# Expression of HAUSP in gliomas correlates with disease progression and survival of patients

**DOI:** 10.3892/or.2013.2342

**Published:** 2013-03-12

**Authors:** CHUANDONG CHENG, CHAOSHI NIU, YANG YANG, YANG WANG, MANMAN LU

**Affiliations:** 1 Department of Neurosurgery, Anhui Provincial Hospital Affiliated to Anhui Medical University, Hefei, Anhui 230001, P.R. China; 2 Anhui Provincial Stereotactic Neurosurgical Institute, Hefei, Anhui 230001, P.R. China; 3 Anhui Province Key Laboratory of Brain Function and Brain Disease, Hefei, Anhui 230001, P.R. China; 4 Basic Medical College of Anhui Medical University, Hefei, Anhui 230001, P.R. China; 5 School of Health Administration, Anhui Medical University, Hefei, Anhui 230032, P.R. China

**Keywords:** glioma, HAUSP/USP7, deubiquitinating enzyme, prognosis

## Abstract

The human herpesvirus-associated ubiquitin-specific protease (HAUSP) deubiquitinating enzyme has been shown to regulate many proteins involved in the cell cycle, as well as tumor suppressors and oncogenes. However, the expression pattern of HAUSP in glioma patients is still unclear. The purpose of the present study was to investigate the expression pattern and prognostic significance of HAUSP in patients with glioma. Eighty glioma specimens and 10 normal control samples were obtained. Immunohistochemical assay, quantitative real-time PCR and western blot analysis were carried out to explore the expression of HAUSP. Additionally, the association of HAUSP expression with clinicopathological parameters and the survival of glioma patients were analyzed. Our results showed that HAUSP expression levels were increased from grade I to grade IV in the tumors of the glioma patients. Moreover, the survival rate of patients with HAUSP-positive tumors was lower when compared to that of patients with HAUSP-negative tumors. We further confirmed that high expression of HAUSP was a significant and independent prognostic indicator in glioma by multivariate analysis. Our data provide convincing evidence for the first time that the overexpression of HAUSP at the gene and protein levels is correlated with poor outcome in patients with glioma in China. HAUSP may play an important oncogenic role in glioma progression, and it is a potential diagnostic and therapeutic target.

## Introduction

Glioma is the most common primary intracranial tumor in both adults and children. The World Health Organization (WHO) classification scheme divides gliomas into grades I through IV, based on increasing levels of malignancy (1). The prognosis of patients with glioma is poor and is closely related to WHO grade. Glioblastoma multiforme (GBM) WHO grade IV is the most malignant variant with a median survival time of 1 year (2). Efforts to better understand the biological basis of glioma progression may yield important, clinically relevant insights into disease management. Many aggressive treatment approaches, such as postoperative chemotherapy and radiation therapy, have been used clinically. Yet, these approaches do not benefit all patients equally. Adverse effects associated with these approaches dramatically deteriorate the quality of life of certain patients. Therefore, individualized therapy should be considered as a valuable approach for patients with high-grade gliomas. Recently, molecular diagnostics has emerged as a powerful tool to discover new biomarkers, network and therapeutic targets, realizing the proof of principle that personalized medicine can increase survival and cure cancer patients. Thus, elucidation of these critical molecular events may improve therapy and individualize therapeutic interventions for patients with gliomas.

Deubiquitylating enzymes (DUBs) antagonize the ubiquitylation of substrates by cleaving polyubiquitin and monoubiquitin and thus afford an additional level of protein post-translational regulation (3). Herpesvirus-associated ubiquitin-specific protease (HAUSP, also known as ubiquitin specific protease 7, USP7) is a cysteine protease that was originally identified as a binding partner for the Herpes simplex viral (HSV) protein infected cell protein 0 (ICP0/Vmw110) (4). Subsequently, numerous proteins have been identified as potential substrate/binding partners of HAUSP (5,6). Some of the better characterized substrates of HAUSP play crucial roles in tumor suppression, DNA repair, immune responses, viral replication and epigenetic control (7). HAUSP has been shown to inactivate several tumor suppressors by nuclear export forkhead box O transcription factor (FOXO4) and phosphatase and tensin homologue deleted on chromosome 10 (PTEN) inactivation (8,9) or degradation [p53 degradation following murine double minute 2 (Mdm2) stabilization] (10,11).

HAUSP is overexpressed in human prostate cancer, and more importantly, high levels of HAUSP are directly correlated with tumor aggressiveness (8). In contrast, Masuya *et al*(12) found that a reduction in HAUSP gene expression may play an important role in NSCLC carcinogenesis, particularly in adenocarcinomas, through p53-dependent pathways. These data suggest that HAUSP may in some instances act as a tumor suppressor (by stabilizing p53) or an oncogene (by stabilizing Mdm2 and redistributing PTEN). Thus, this regulation may be dependent on genetic context and also tissue or cell type due to differential expression of other proteins.

In order to gain further insight into the role of HAUSP in the progression of glioma, we used immunohistochemical assay, quantitative real-time PCR and western blot analysis to investigate the expression pattern of HAUSP in glioma specimens and normal control brain tissues. Next, we analyzed the relationship between HAUSP expression and the glioma stage as well as the survival of patients.

## Materials and methods

### Patients and clinical specimens

This study was approved by the Research Ethics Committee of Anhui Provincial Hospital of Anhui Medical University. Written informed consent was obtained from all of the patients. All specimens were handled and anonymized according to ethical and legal standards.

Fresh glioma specimens were obtained from 80 patients who underwent surgical treatment at the Department of Neurosurgery, Anhui Provincial Hospital, Anhui Medical University between January 2008 and October 2008. None of the patients had received radiotherapy, immunotherapy and chemotherapy prior to surgery. Additionally, normal brain tissue samples were obtained from 10 patients who underwent surgery for reasons other than malignancy, such as cerebral trauma. These normal control samples were collected during partial resection of normal brain tissue for decompression treatment following severe head injury. Parts of each specimen were snap-frozen in liquid N_2_ for 10 min and stored in a −80°C ultra-freezer for mRNA and protein isolation; other parts of each specimen were fixed in 10% formalin and paraffin-embedded for histological sectioning. All of the glioma samples were verified by pathological analysis and classified according to the WHO 2007 classification standard. There were 27 low-grade (WHO grades I and II) and 53 high-grade tumors (WHO grades III and IV) (Table I). Patient data included age, gender, date and type of initial operation, and details of the follow-up. Clinical information was obtained by reviewing medical records and radiographic images, by interview in the clinic or by telephone, and by review of death certificate. A patient was considered to have recurrent disease when revealed either by magnetic resonance imaging or the occurrence of new neurologic symptoms. In the follow-up period, overall survival was calculated from diagnosis to death or last follow-up; the total period of follow-up was 16–60 months.

### Immunohistochemical assays

The formalin-fixed, paraffin-embedded tissue sections (4 μm) were deparaffinized in xylene and dehydrated through a graduated alcohol series. Endogenous peroxidase activity was blocked with 3% H_2_O_2_ in methanol for 20 min. Antigen retrieval was performed by microwaving sections in 0.01 M sodium citrate (pH 6.0). Non-specific binding was blocked by incubating sections with 5% BSA in phosphate-buffered saline (PBS) for 30 min at room temperature. Without being washed, these sections were incubated with polyclonal anti-HAUSP antibody (sc-30164; 1:400; Santa Cruz Biotechnology Inc., CA, USA) in PBS at 4°C overnight in a moist box. Wash steps the sections were incubated with biotinylated goat anti-rabbit immunoglobulin G (1:400; Sigma) for 1 h at room temperature wash steps, and expression was detected using the streptavidin-peroxidase complex. The brown color indicative of peroxidase activity was developed by incubation with 0.1% 3,3-diaminobenzi-dine (Sigma) in PBS with 0.05% H_2_O_2_ for 5 min at room temperature. The sections were lightly counterstained with hematoxylin. Two pathologists scored the immunohistochemical staining under double-blinded conditions without prior knowledge of the clinical or clinicopathological status of the specimens. Images captured for all sections were acquired using an Olympus BX51. The expression of HAUSP in glioma tissue was evaluated by scanning the entire tissue specimen under low magnification (x40), followed by confirmation by scanning under high magnification (×200 and ×400). Positive cells were indicated by the presence of a distinct brown color in the nuclei or cytoplasm. Normal brain tissues were used as control tissues, and non-immune IgG was also used as a negative control antibody for immunohistochemical staining.

### Real-time polymerase chain reaction

Total RNA was purified from all 80 glioma specimens and 10 control normal brain tissues using TRIzol reagent (Invitrogen, Carlsbad, CA, USA). The concentration and purity of RNA were determined spectrophotometrically at 260/280 nm using a Nanodrop spectrophotometer (Ocean Optics, Dunedin, FL, USA). Then cDNA was synthesized from ~5 μg RNA/20 μl using a cDNA reverse transcription kit (Qiagen, Germany). Real-time polymerase chain reaction (real-time PCR) amplification was performed using a 7500 Fast System Real-Time PCR cycler (Applied Biosystems, Foster City, CA, USA). Primers were designed using Primer Express v3.0 software (Applied Biosystems). The HAUSP primers were 5′-ATGAACCACCAGCAGCAGCAGC-3′ (forward) and 5′-GCGTGGCATCACCATAATCTTCC-3′ (reverse), while the internal control β-actin primers were 5′-ATGGATGATGATATCGCCGCGCTC-3′ (forward) and 5′-TTTCTCCATGTCGTCCAGTTGG-3′ (reverse). After first-strand synthesis, an equivalent of 50 ng of starting total cellular RNA (1/10 of the cDNA reaction) was added to two duplicate PCR reaction tubes, each containing 12.5 μl SYBR-Green mix, 0.5 μl SYBR-Green Rox (both from Takara, Dalian, Liaoning, China), 100 nmol/l forward primer, and 100 nmol/l reverse primer in a final volume of 25 μl. The amplification protocol consisted of denaturation at 95°C for 10 min (to activate the enzyme), followed by 45 cycles of denaturation at 95°C for 10 sec and annealing and extension at 60°C for 34 sec, using an ABI SDS 7500 system (Applied Biosystems). Expression of HAUSP was analyzed using the 2^−ΔΔCt^ method. HAUSP mRNA expression was normalized relative to expression of β-actin in the same sample.

### Western blot analysis

Total tissue proteins were purified from all 80 glioma tissue samples and 10 normal brain tissue specimens and were lysed in SDS lysis buffer (50 mM Tris/HCl pH 8.0, 150 mM NaCl, 1 mM EDTA, 0.5% SDS), followed by centrifugation at 12,000 × g for 10 min at 4°C. The supernatants were collected and protein concentrations were determined using a Bio-Rad protein assay dye reagent (Bio-Rad, Hercules, CA, USA). For electrophoresis, aliquots containing equal amounts of whole protein lysate (50 μg) were separated by size on 4–20% polyacrylamide gel (Invitrogen) under SDS denaturing conditions. Separated proteins were transferred to PVDF membranes at 200 mA for 2 h. The membranes were blocked with 5% non-fat milk in 1X Tris-buffered saline containing 0.1% Tween-20 and incubated with primary antibody against HAUSP (sc-30164; 1:1,000; Santa Cruz Biotechnology Inc.), or actin (1:1,000; Beyotime, China) overnight at 4°C. Finally, blots were incubated with horseradish peroxidase (HRP)-conjugated goat anti-rabbit or goat anti-mouse IgG antibody (ZSGB-BIO) for 1 h at room temperature. Immunoblots were visualized by chemiluminescence using an ECL detection system (BeyoEcl Plus; Beyotime) and the intensity of the bands was determined using the Image-Pro Plus 6.0 software (Japan).

### Statistical analysis

All statistical analyses were performed using SPSS software (version 16.0; SPSS Inc., Chicago, IL, USA). The rank-sum test was used to analyze ranked data. Measured data were analyzed using one-way analysis of variance (ANOVA). Randomized block design ANOVA was used to analyze the differences between different tissue types. In the analysis of glioma morbidity for all patients, we used the Kaplan-Meier estimator and univariate Cox regression analysis to assess the marginal effect of each factor. The differences between groups were tested by log-rank analyses. The joint effect of different factors was assessed using multivariate Cox regression. Spearman’s analysis was carried out to analyze the correlation between HAUSP mRNA and protein expression levels. A P-value <0.05 was considered to indicate a statistically significant result.

## Results

### Expression levels of HAUSP in patients with malignant gliomas and normal brain tissue specimens by immunohistochemical assay and survival analysis

**Figure 1:**
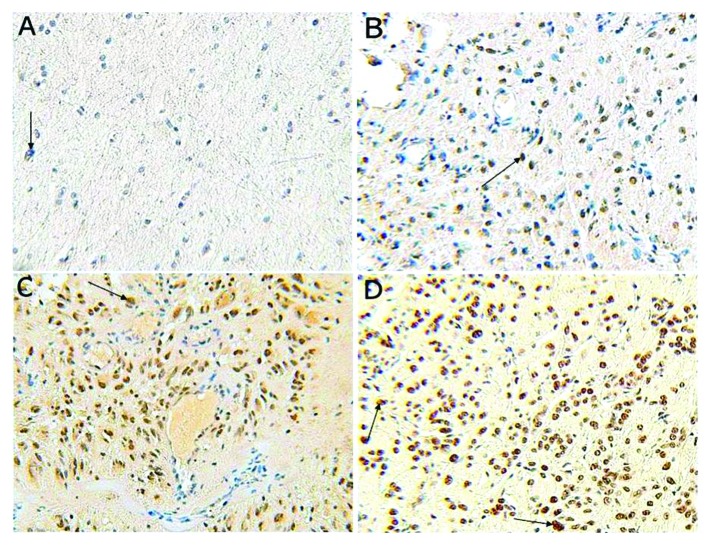
Immunohistochemical staining of HAUSP protein in tumor cells of different World Health Organization grades [(B) WHO II; (C) WHO III; (D) WHO IV] and normal brain tissue (A) as control (original magnification, ×200). Staining for this antigen is described in Materials and methods. Positive staining of HAUSP was noted predominantly in the nuclei of tumors cells, but a weaker cytoplasmic reaction was also observed. HAUSP protein was more abundant in (C and D) the high-grade when compared with that in the (B) low-grade tumors. (A) Nearly negative expression of HAUSP was observed in the normal brain tissues. Arrows indicate the positive staining of HAUSP in glioma and normal brain tissues.

HAUSP expression was assessed in a total of 80 glioma specimens of which 27 were low-grade glioma (grades I and II) and 53 were high-grade (grades III and IV). Ten specimens obtained from normal brain tissue served as the control group. Based on immunohistochemical analysis, positive staining for HAUSP was predominantly localized in the nuclei of tumor cells, but a weaker cytoplasmic reaction was noted (Fig. 1). Among the glioma specimens, 69 (86.25%) glioma specimens were positively stained, and 11 (13.75%) glioma specimens were negatively stained. Among the control specimens, 6 (60.0%) were positively stained with only weak staining observed in a few cell nuclei, and 4 (40.0%) were negatively stained. We also found a significant increase in HAUSP expression in glioma when compared with the normal brain tissues (P<0.05).

Based on the hierarchical scores of the immunohistochemical staining, we analyzed the relationship between HAUSP staining and clinical factors. HAUSP expression was not significantly affected by gender and age (both P>0.05) of the patients. In contrast, the HAUSP expression was closely associated with WHO grade, with expression increasing from grade I to IV (P<0.05; Table II). We also found that HAUSP protein expression was higher in patients with Karnofsky performance status (KPS) <80 than in those with KPS ≥80 (P<0.05; Table II).

**Figure 2:**
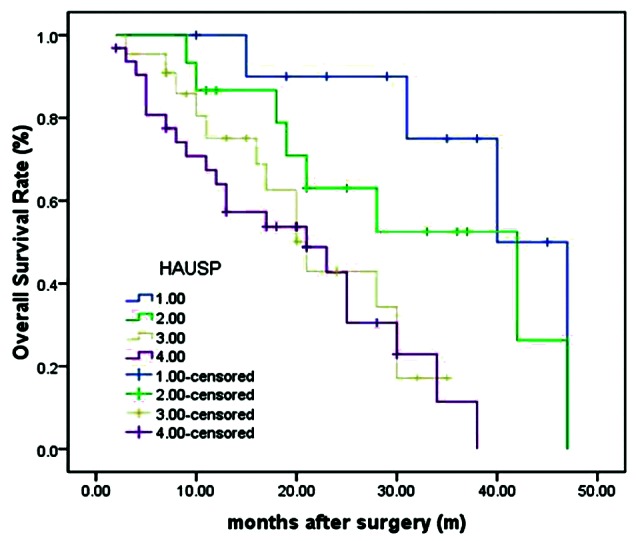
Kaplan-Meier curves indicating the overall survival rates in the glioma patients subgrouped according to HAUSP protein expression (1, −; 2, +; 3, ++; 4, +++).

Moreover, we reviewed clinical information of the glioma patients with HAUSP-positive or -negative tumors. During the follow-up period, 61 of the 80 glioma patients (76.25%) succumbed to disease (57 from the HAUSP-positive group and 4 from the HAUSP-negative group). As determined by the log-rank test, the survival rate of patients with tumors lacking HAUSP staining was longer than those with HAUSP-positive staining tumors (P<0.001) (Fig. 2). The median survival time of patients with negative expression of HAUSP could not be estimated by statistical analysis since all patients survived longer than the overall median level, and median survival time of patients with tumors with strong positive (+++), moderate positive (++) and weak positive (+) of HAUSP were 7.9±0.6, 12.8±1.2 and 21.5±2.1 months (log-rank test, P=0.003).

Furthermore, the post-operative survival curve of patients with glioma and HAUSP expression after adjusting for age, gender, WHO grade and KPS was plotted. By multivariate analysis, overexpression of HAUSP was a significant and independent prognostic indicator for patients with glioma besides age, WHO grade and KPS. The Cox proportional hazards model showed that higher HAUSP expression was associated with poor overall survival.

### Quantitative analysis of HAUSP mRNA expression in glioma by RT-PCR

**Figure 3:**
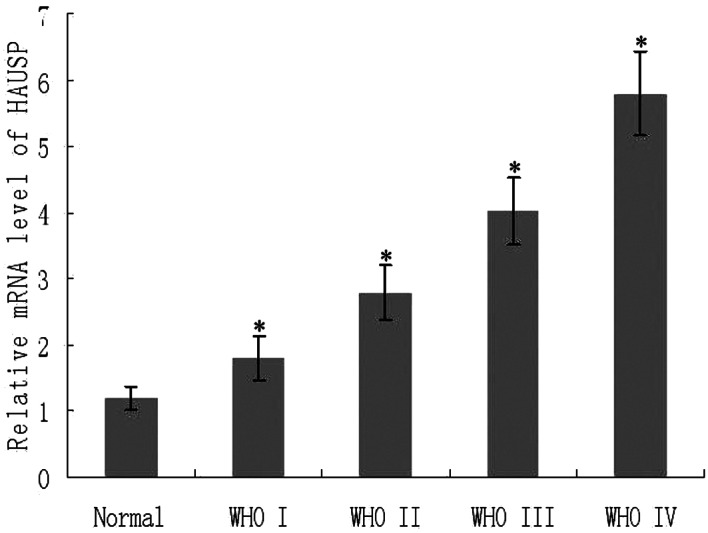
Relative mRNA level of HAUSP expression in glioma of different World Health Organization grades and normal brain tissue as determined by real-time PCR (^*^P<0.05, as estimated using complete block design analysis of variance).

We determined the mRNA expression of HAUSP normalized to β-actin by real-time PCR. As shown in Fig. 3, there was an obvious increase in the expression of HAUSP mRNA from the control normal brain tissues to glioma tissues (P<0.05). We further analyzed the expression of HAUSP mRNA based on KPS and WHO grade. Notably, HAUSP mRNA expression increased in patients whose KPS was <80 (P<0.001) and also increased with advancement of WHO grade I to IV (P<0.01). There was a significant positive correlation between the expression of HAUSP mRNA and protein expression levels from the same glioma tissues (rs=0.878, P<0.001).

### Quantitative analysis of HAUSP protein expression based on WHO grade in gliomas by western blotting

**Figure 4:**
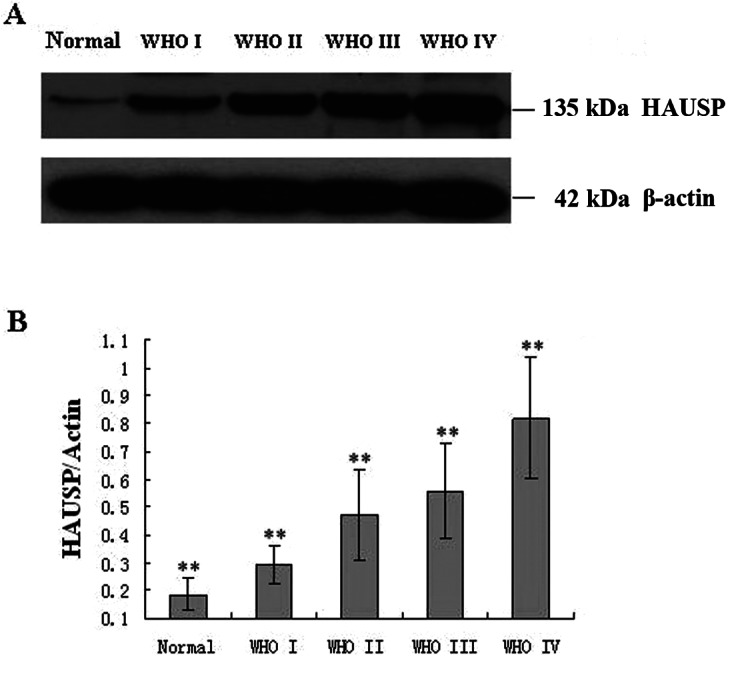
HAUSP protein expression in glioma as determined by western blotting. (A) HAUSP protein expression in representative gliomas of different World Health Organization grades and normal brain tissue (as control), relative to actin expression (loading control). (B) Mean expression of HAUSP in gliomas of each grade and normal brain tissue, as determined by densitometric analysis of western blotting, normalized against expression of actin as a loading control (n=90, ^**^P<0.05, as estimated using Student’s t-test). Error bars, ± SD.

Based on the results of western blot analysis, we found that HAUSP protein expression tended to increase from the normal brain tissue to the glioma. We also investigated whether the expression of HAUSP correlated with WHO grade. HAUSP expression was lowest in grade I and highest in grade IV (Fig. 4). This result agreed with the findings of the immunohistochemical analysis and indicated a close correlation of HAUSP protein expression with WHO grade.

## Discussion

In the present study, we investigated the expression of HAUSP in 80 cases of human glioma and 10 cases of human normal brain tissues, and compared the expression with tumor grade and the survival rates of patients. Our data demonstrated that HAUSP protein was overexpressed in glioma compared to that in the normal brain tissue. HAUSP mRNA expression was also increased in glioma compared with the control normal brain tissue. We found an increasing trend of both HAUSP protein and mRNA levels from WHO grade I to WHO grade IV glioma. These results suggest that the transcriptional activation of human HAUSP may participate in the carcinogenesis and progression of glioma. HAUSP may have an important role during the genesis or progression of glioma.

Protein degradation in eukaryotic cells is mediated by either the lysosome or ubiquitin-proteasome pathway (13). Ubiquitination and deubiquitination have a precise role for selective protein degradation in eukaryotic cells, and are important for the regulation of a number of intracellular processes including cell cycle, apoptosis, transcriptional activation, signal transduction, antigen presentation, oncogenesis, preimplantation, and DNA repair (3,14,15). Deregulation of the ubiquitin-proteasome system has been implicated in the pathogenesis of many human diseases, including cancer.

Ubiquitylation, the covalent attachment of one or more units of the 76-amino acid peptide ubiquitin, is an important post-translational modification that regulates the levels, activity and localization of many cellular proteins (16). Ubiquitylation is a reversible modification; ubiquitin can also be removed from substrates by DUBs (17). By specifically removing ubiquitin chains, DUBs modulate the ubiquitylation status of a variety of proteins, and thereby contribute to the regulation of important cellular processes, such as the regulation of the substrate protein nucleocytoplasmic distribution (18), gene expression (19), proliferation (20), repair of DNA damage (21) and apoptosis (22). Of the DUBs, USP7, also known as herpes virus-associated ubiquitin-specific protease HAUSP, has been well characterized.

HAUSP is a 135-kDa protein in the USP family of the DUB enzymes. It is primarily a nuclear protein and localizes to a subset of PML bodies (23). In addition to containing a DUB domain, it also has a C-terminal domain that contains at least five ubiquitin-like domains (24) and an N-terminal TRAF-like MATH domain (25). It is produced ubiquitously and is an evolutionarily highly conserved protein in eukaryotes, which was originally isolated as a binding partner of the herpes simplex virus protein Vmw110/ICP0 (4).

A number of studies have indicated, at the molecular level, by virtue of its deubiquitinating activity, that HAUSP regulates the steady-state level of several polyubiquitinated substrates. For example, HAUSP modulates the level of the p16^INK4a^ and p53 tumor suppressors through changes in the stabilization of Bmi1/Mel18 and Mdm2, respectively (10,11,26). HAUSP binding to p53 was recently shown to be regulated by TSPYL5, a protein potentially involved in breast oncogenesis through its competition with p53 for binding to the same region of HAUSP (27). Additional proteins involved in genomic integrity and regulation, such as the DNMT1 DNA methylase and the claspin adaptor, are also stabilized by HAUSP through deubiquitination (28,29). HAUSP was shown to regulate the cellular compartmentalization of several monoubiquitinated substrates by deubiquitination. Concerning this point, in response to oxidative stress, HAUSP inhibits nuclear localization and transcriptional activity of the forkhead box O transcription factor (FOXO4) by interacting with and deubiquitylating FOXO protein (9). Through a similar mechanism, HAUSP deubiquitylates tumor suppressor PTEN, and thus regulates its nuclear exclusion (8). HAUSP overexpression in human prostate cancer was directly associated with tumor aggressiveness, most likely through PTEN mislocalization (8). Previous *in vivo* data also underlined the involvement of HAUSP in cancer cell proliferation (30). Therefore, HAUSP exerts both p53-dependent and p53-independent effects on controlling cell proliferation and apoptosis. Collectively, the connections between HAUSP and several pathways involving oncogenes and tumor suppressors, strongly suggest that it may play a role in the carcinogenesis of different types of tumor.

The present results confirm that HAUSP is overexpressed during glioma progression. We further analyzed the correlation of HAUSP expression and survival rates of patients. Our data revealed that only 13.75% (11/80) of glioma cases showed negative staining for HAUSP. The survival rate of patients with strong positive or moderate positive staining tumors for HAUSP was lower than the survival rates of patients with tumors showing HAUSP-negative or weak positive staining. Kaplan-Meier analysis of the survival curves showed a significantly worse overall survival for patients whose tumors had high HAUSP levels, indicating that high HAUSP protein level is a marker of poor prognosis for patients with glioma in our study. Moreover, multivariate analysis showed that overexpression of HAUSP may be a marker of worse outcome independent of known clinical prognostic indicators such as age, KPS and grade. These data indicate that high expression of HAUSP is correlated with a worse outcome of patients with glioma in China. Thus, HAUSP may be an independent predictor of survival for glioma patients. In our study, which consisted of 90 cases of glioma and normal brain samples, HAUSP expression was analyzed by immunohistochemistry, real-time PCR and western blot analysis. Thus, using a comprehensive methodology and a detailed clinical follow-up in our study the results were reliable.

A recent study revealed that HAUSP counterbalances REST (also known as neuron restrictive silencer factor, NRSF) ubiquitination and prevents nasopharyngeal carcinoma (NPC) differentiation. HAUSP expression was found to decline concordantly with REST upon neuronal differentiation and reciprocally with β-TrCP levels. HAUSP knockdown in NPCs decreased REST and induced differentiation. In contrast, HAUSP overexpression upregulated REST by overriding β-TrCP-mediated ubiquitination. Furthermore, REST overexpression in NPCs rescued the differentiation phenotype induced by HAUSP knockdown. It demonstrated that HAUSP stabilized REST through deubiquitination and antagonized β-TrCP in regulating REST at the post-translational level. Thus, the HAUSP-mediated deubiquitination represents a critical regulatory mechanism involved in the maintenance of NPCs. Expression of functional HAUSP is critical for preventing REST ubiquitination and suppressing NPC differentiation. All-*trans* retinoic acid (RA) induced NPCs to undergo cellular differentiation, HAUSP and REST protein levels gradually decreased during NPC differentiation. HAUSP may play critical roles in the stabilization of stem cell transcription factors and may promote maintenance of ‘stemness’ (31).

Embryonic stem cells (ESCs) and cancer cells share many key biological properties, such as self-renewal, an undifferentiated state, extensive proliferative potency, pluripotency and differentiation capacity. These parallel features suggest that similar mechanisms may be involved in regulating ESCs and cancer cells. The cancer stem cell (CSC) theory of tumorigenesis assumes the possibility of the identification of a small group of tumor cells responsible for the occurrence, growth, and recurrence of tumors in different types of cancers including gliomas (32–34). Transcriptional regulators of stem cell maintenance and differentiation require exquisite control to direct cell fate determination. Uncontrolled activation of core stem cell pathways drives transformation while loss of function in these cellular mechanisms leads to degenerative conditions (35). A number of transcriptional factors (Nanog, c-Myc, Sox2 and Oct3/4) serving as core regulators of self-renewal and maintenance of ESCs and tissue stem cells have been found to be ubiquitylated by different E3 ubiquitin ligases. It is likely that each stem cell transcription factor can be deubiquitylated by a specific deubiquitylase (35), and different types of transcriptional factors play a key role in the pathogenesis of gliomas and maintain the undifferentiated state of glioma cells. For example, our previous study indicated that the ESC transcriptional factor, NANOG, was overexpression in glioma tissues when compared with that in normal brain tissues at the mRNA and protein levels. An association between higher NANOG expression and aggressive grades of gliomas was also demonstrated. It may contribute to the existence of BTSCs and may be related to tumorigenesis of the cerebrum by maintaining the undifferentiated state of glioma cells (36). We speculate that overexpression of HAUSP in gliomas may be involved in a network that counterbalances transcriptional factor ubiquitination, stabilizes stem cell transcription factors, prevents glioma cell differentiation and promotes maintenance of ‘stemness’.

In conclusion, our comprehensive analysis indicated that overexpression of HAUSP appears to be intimately involved in the pathogenesis of gliomas, and the activity of HAUSP may contribute to maintaining an undifferentiated state of glioma cells. On the basis of these findings, we assume that inhibition of HAUSP, important in the progression of glioma cell transformation, may block the tumorigenesis of gliomas, and targeting HAUSP may be an approach to improve the therapeutic intervention for poorly differentiated gliomas. However, further study is required to determine the precise role of HAUSP and the mechanism of HAUSP transcriptional regulation in gliomas, in particular, the relationship between the transcriptional factors of ESCs that are involved in the pathogenesis of gliomas.

## Figures and Tables

**Table I:** Level of expression of HAUSP protein in glioma specimens as determined using immunohistochemical analysis, and the comparison with clinicopathological variables.

		Expression of HAUSP protein				
			
	n	−	+	++	+++	P-value
Total	80	11	15	22	32	
Gender						0.925^a^
Male	48	7	9	15	17	
Female	32	4	6	7	15	
Age (years)						0.378^a^
<60	54	6	10	16	21	
≥60	26	5	5	6	10	
KPS						<0.05^a^
<80	47	4	8	14	21	
≥80	33	7	7	8	11	
WHO grade						<0.05^b^
I	8	3	4	1	0	
II	19	4	5	7	3	
III	25	3	3	8	11	
IV	28	1	3	6	18	

−, negative staining; +, weak positive staining; ++, moderate positive staining; +++, strong positive staining.

a P-value was estimated using the Mann-Whitney U-test;

b P-value was estimated using the Kruskal-Wallis test.

HAUSP, herpesvirus-associated ubiquitin-specific protease; KPS, Karnofsky performance scale; WHO, World Health Organization.

**Table II:** Mean values of HAUSP mRNA expression in clinical glioma samples and normal control tissues, and comparison with clinicopathological variables.

	n	HAUSP expression (mean ± SD)	P-value
Tissue type			<0.05^a^
Glioma	80	3.5776±0.6498	
Normal control	10	1.1928±0.1821	
Gender			0.392^a^
Male	48	3.5152±0.5682	
Female	32	3.6686±0.5147	
Age (years)			0.213^a^
<60	54	3.4996±0.4941	
≥60	26	3.7258±0.5266	
KPS			<0.05^a^
<80	43	3.7128±0.5182	
≥80	37	3.4245±0.4814	
WHO grade			<0.05^b^
I	8	1.7918±0.3281	
II	19	2.7768±0.4146	
III	25	4.0352±0.5063	
IV	28	5.7928±0.6225	

a P-value was estimated using Student’s t-test;

b P-value was estimated using complete block design analysis of variance.

HAUSP, herpesvirus-associated ubiquitin-specific protease; KPS, Karnofsky performance scale; WHO, World Health Organization.

## References

[1] Louis DN, Ohgaki H, Wiestler OD (2007). The 2007 WHO classification of tumours of the central nervous system. Acta Neuropathol.

[2] Van Meir EG, Hadjipanayis CG, Norden AD, Shu HK, Wen PY, Olson JJ (2010). Exciting new advances in neuro-oncology: the avenue to a cure for malignant glioma. CA Cancer J Clin.

[3] Hershko A, Ciechanover A (1998). The ubiquitin system. Annu Rev Biochem.

[4] Everett RD, Meredith M, Orr A, Cross A, Kathoria M, Parkinson J (1997). A novel ubiquitin-specific protease is dynamically associated with the PML nuclear domain and binds to a herpesvirus regulatory protein. EMBO J.

[5] Sowa ME, Bennett EJ, Gygi SP, Harper JW (2009). Defining the human deubiquitinating enzyme interaction landscape. Cell.

[6] Kessler BM, Fortunati E, Melis M, Pals CE, Clevers H, Maurice MM (2007). Proteome changes induced by knockdown of the deubiquitylating enzyme HAUSP/USP7. J Proteome Res.

[7] Nicholson B, Suresh Kumar KG (2011). The multifaceted roles of USP7: new therapeutic opportunities. Cell Biochem Biophys.

[8] Song MS, Salmena L, Carracedo A, Egia A, Lo-Coco F, Teruya-Feldstein J, Pandolfi PP (2008). The deubiquitinylation and localization of PTEN are regulated by a HAUSP-PML network. Nature.

[9] van der Horst A, de Vries-Smits AM, Brenkman AB, van Triest MH, van den Broek N, Colland F, Maurice MM, Burgering BM (2006). FOXO4 transcriptional activity is regulated by monoubiquitination and USP7/HAUSP. Nat Cell Biol.

[10] Cummins JM, Rago C, Kohli M, Kinzler KW, Lengauer C, Vogelstein B (2004). Tumour suppression: disruption of HAUSP gene stabilizes p53. Nature.

[11] Li M, Brooks CL, Kon N, Gu W (2004). A dynamic role of HAUSP in the p53-Mdm2 pathway. Mol Cell.

[12] Masuya D, Huang C, Liu D, Nakashima T, Yokomise H, Ueno M, Nakashima N, Sumitomo S (2006). The HAUSP gene plays an important role in non-small cell lung carcinogenesis through p53-dependent pathways. J Pathol.

[13] Glickman MH, Ciechanover A (2002). The ubiquitin-proteasome proteolytic pathway: destruction for the sake of construction. Physiol Rev.

[14] Ciechanover A (1998). The ubiquitin-proteasome pathway: on protein death and cell life. EMBO J.

[15] Baek KH (2003). Conjugation and deconjugation of ubiquitin regulating the destiny of proteins. Exp Mol Med.

[16] Grabbe C, Husnjak K, Dikic I (2011). The spatial and temporal organization of ubiquitin networks. Nat Rev Mol Cell Biol.

[17] Komander D, Clague MJ, Urbé S (2009). Breaking the chains: structure and function of the deubiquitinases. Nat Rev Mol Cell Biol.

[18] García-Santisteban I, Banuelos S, Rodríguez JA (2012). A global survey of CRM1-dependent nuclear export sequences in the human deubiquitinase family. Biochem J.

[19] Frappier L, Verrijzer CP (2011). Gene expression control by protein deubiquitinases. Curr Opin Genet Dev.

[20] Song L, Rape M (2008). Reverse the curse - the role of deubiquitination in cell cycle control. Curr Opin Cell Biol.

[21] Bergink S, Jentsch S (2009). Principles of ubiquitin and SUMO modifications in DNA repair. Nature.

[22] Ramakrishna S, Suresh B, Baek KH (2011). The role of deubiquitinating enzymes in apoptosis. Cell Mol Life Sci.

[23] Muratani M, Gerlich D, Janicki SM, Gebhard M, Eils R, Spector DL (2002). Metabolic-energy-dependent movement of PML bodies within the mammalian cell nucleus. Nat Cell Biol.

[24] Faesen AC, Dirac AM, Shanmugham A, Ovaa H, Perrakis A, Sixma TK (2011). Mechanism of USP7/HAUSP activation by its C-terminal ubiquitin-like domain and allosteric regulation by GMP-synthetase. Mol Cell.

[25] Zapata JM, Pawlowski K, Haas E, Ware CF, Godzik A, Reed JC (2001). A diverse family of proteins containing tumor necrosis factor receptor-associated factor domains. J Biol Chem.

[26] Maertens GN, El Messaoudi-Aubert S, Elderkin S, Hiom K, Peters G (2010). Ubiquitin-specific proteases 7 and 11 modulate Polycomb regulation of the INK4a tumour suppressor. EMBO J.

[27] Epping MT, Meijer LA, Krijgsman O, Bos JL, Pandolfi PP, Bernards R (2010). TSPYL5 suppresses p53 levels and function by physical interaction with USP7. Nat Cell Biol.

[28] Du Z, Song J, Wang Y (2010). DNMT1 stability is regulated by proteins coordinating deubiquitination and acetylation-driven ubiquitination. Sci Signal.

[29] Faustrup H, Bekker-Jensen S, Bartek J, Lukas J, Mailand N (2009). USP7 counteracts SCFbetaTrCP- but not APCCdh1-mediated proteolysis of Claspin. J Cell Biol.

[30] Becker K, Marchenko ND, Palacios G, Moll UM (2008). A role of HAUSP in tumor suppression in a human colon carcinoma xenograft model. Cell Cycle.

[31] Huang Z, Wu Q, Guryanova OA, Cheng L, Shou W, Rich JN, Bao S (2011). Deubiquitylase HAUSP stabilizes REST and promotes maintenance of neural progenitor cells. Nat Cell Biol.

[32] Singh SK, Clarke ID, Terasaki M, Bonn VE, Hawkins C, Squire J, Dirks PB (2003). Identification of a cancer stem cell in human brain tumors. Cancer Res.

[33] Bertrand J, Begaud-Grimaud G, Bessette B, Verdier M, Battu S, Jauberteau MO (2009). Cancer stem cells from human glioma cell line are resistant to Fas-induced apoptosis. Int J Oncol.

[34] Li G, Chen Z, Hu YD, Wei H, Li D, Ji H, Wang DL (2009). Autocrine factors sustain glioblastoma stem cell self-renewal. Oncol Rep.

[35] Huang Z, Zhou W, Bao S (2011). Role of deubiquitylase HAUSP in stem cell maintenance. Cell Cycle.

[36] Niu CS, Li DX, Liu YH, Fu XM, Tang SF, Li J (2011). Expression of NANOG in human gliomas and its relationship with undifferentiated glioma cells. Oncol Rep.

